# Comparison of Various Equations for Estimating GFR in Malawi: How to Determine Renal Function in Resource Limited Settings?

**DOI:** 10.1371/journal.pone.0130453

**Published:** 2015-06-17

**Authors:** Nicola Glaser, Andreas Deckert, Sam Phiri, Dietrich Rothenbacher, Florian Neuhann

**Affiliations:** 1 Institute of Public Health, University of Heidelberg, Heidelberg, Germany; 2 The Lighthouse Trust, Lilongwe, Malawi; 3 Department of Medicine, University of North Carolina, Chapel Hill, North Carolina, United States of America; 4 Institute of Epidemiology and Medical Biometry, Ulm University, Ulm, Germany; Rouen University Hospital, FRANCE

## Abstract

**Background:**

Chronic kidney disease (CKD) is a probably underrated public health problem in Sub-Saharan-Africa, in particular in combination with HIV-infection. Knowledge about the CKD prevalence is scarce and in the available literature different methods to classify CKD are used impeding comparison and general prevalence estimates.

**Methods:**

This study assessed different serum-creatinine based equations for glomerular filtration rates (eGFR) and compared them to a cystatin C based equation. The study was conducted in Lilongwe, Malawi enrolling a population of 363 adults of which 32% were HIV-positive.

**Results:**

Comparison of formulae based on Bland-Altman-plots and accuracy revealed best performance for the CKD-EPI equation without the correction factor for black Americans. Analyzing the differences between HIV-positive and –negative individuals CKD-EPI systematically overestimated eGFR in comparison to cystatin C and therefore lead to underestimation of CKD in HIV-positives.

**Conclusions:**

Our findings underline the importance for standardization of eGFR calculation in a Sub-Saharan African setting, to further investigate the differences with regard to HIV status and to develop potential correction factors as established for age and sex.

## Introduction

Chronic kidney disease (CKD) constitutes a leading cause of morbidity and mortality in high income countries and is increasingly recognized as important for low and middle income countries (LMICs). [1,2] The impact in LMICs is aggravated by the combination of increasing non-communicable diseases (NCDs) with the continuing burden of infectious diseases and limited access to health care services. [3–5] However, knowledge about the prevalence of CKD in sub-Saharan-Africa (SSA) still remains limited. Reliable data sources on morbidity and mortality such as death registers are not available. [6,7] Estimates suggest about 200–300 per million people are living with CKD in SSA. [8]

The few published studies show a large variation of CKD prevalence ranging from 4.7% in HIV-negatives in Uganda [9] up to 33.5% in HIV-positives in Zambia. [10] When defining CKD by proteinuria or eGFR <60ml/min/1.73 m², a recently published systematic review reported an average prevalence of CKD in SSA of 13.9%. [11] A study conducted in Kinshasa, DRC, found 12.4% prevalence of CKD estimated by the MDRD-equation. Hypertension and age were independently associated with CKD stage 3 and hypertension also with proteinuria. [12] As HIV still constitutes a major public health problem in SSA and can itself cause nephropathy, there is more data about CKD in HIV-positive than HIV-negative individuals. [13–15] Reported large variations in prevalence of CKD in SSA may result from different thresholds used for the definition of CKD, differences in study design, or non-comparability of the equations and laboratory methods applied to estimate renal function. [16]

The increasing relevance of chronic non-communicable diseases in regions like SSA emphasizes the need to establish appropriate and well validated methods to assess renal function. Guidelines developed by the American Kidney Foundation promote the use of creatinine based equations to estimate the glomerular filtration rate (GFR), [17] such as the Cockcroft-Gault-formula, [18] and the MDRD-4, [19] and in 2012, the Kidney Diseases Improving Global Outcomes (KDIGO) organization [20] recommended the use of the CKD-EPI-formula. [21] Creatinine-based equations are preferred as the serum creatinine determination is a simple, non-expensive, internationally standardized test. [22] Nonetheless, these equations have originally been developed and evaluated in Northern American patient cohorts with mild CKD and therefore might not simply be applicable to SSA cohorts. [21]

Serum creatinine depends on various factors such as sex, age, muscle mass, nutrition and physical activity, [21] some of which are linked to socioeconomic status. Therefore, a noncritical application of these formulae in low and middle income countries appears questionable. Levey et al. found significant differences in the serum creatinine levels of self-defined white and black Americans, which were not related to the measured eGFR. Hence, to adjust the eGFR for these differences in serum creatinine, Levey et al. established a correction factor for black Americans for the MDRD and CKD-EPI GFR estimation formulas. [19,21] However, the differences attributed to “black American” could be confounded by the socioeconomic class factors in the US or epi-genetic adaptations due to the history of slavery in the US. [23–25] A study conducted in Ghana showed that GFR calculated from 24 hour urine collection was best comparable to eGFR either by MDRD-4 or CKD-EPI omitting the factor for black Americans. [26] In many studies previously conducted in SSA it is not evident whether this factor, which is often incorrectly referred to as a correction factor for black skin colour, was applied or not.

Another approach to estimate kidney function is using cystatin C based equations. Cystatin C is produced at a relatively constant rate, and is not significantly influenced by inflammatory processes. [27] Cystatin C depends less on body characteristics such as muscle mass and is suggested to better estimate the GFR compared to serum creatinine based equations, even in HIV positives. Various studies show a stronger correlation of gold-standard GFR and serum cystatin C estimated GFR compared to serum creatinine estimated GFR, especially in HIV-positives. [28,29] Nevertheless in the foreseeable future Cystatin C estimates will not be available in most of the SSA laboratories. Reliable estimates of CKD prevalence in SSA regions in order to guide treatment and prevention strategies will require the development of a standardized, possibly creatinine-based GFR-estimation formula. We used data of HIV-positive adults not on antiretroviral treatment and HIV-negative adults as part of a study at a HIV-testing centre in central Malawi to validate the performance of various creatinine-based estimating equations of GFR in comparison to a cystatin C formula.

## Methods

### Study design and study population

Between the 24^th^ of January 2012 and the 29^th^ of March 2012 a cross-sectional survey was conducted to analyse the prevalence of renal impairment in the study population and to assess and compare the diagnostic validity of the different GFR estimation formulae. All individuals over 18 years of age and ART-naïve, coming to the HIV counselling and testing centre at the Lighthouse Clinic in Lilongwe, Malawi were invited to participate in the study. No other exclusion criteria applied. This large HIV clinic serves a mainly urban catchment population of altogether 1,9 million people. [30] Following informed consent, a standardized questionnaire about age, gender, possible pregnancy, current symptoms, medical and family history was administered. Body height and weight were taken in a standardized way and blood pressure was measured using the same calibrated standard automatic blood pressure device (Omron, Germany) on the free right arm at heart level after at least 10 minutes of sitting with the back at the backrest of the chair.

### Laboratory measurements

#### Assessment of renal function

Following venous blood draw and centrifugation an aliquot of serum was frozen at -80° Celsius and shipped to Germany (dry ice). Serum creatinine and cystatin C were analysed at University of Heidelberg. Serum creatinine was determined with a photometric measurement and traceable to an isotope dilution mass spectrometry (IDMS) reference measurement procedure, [22] according to standards. Cystatin C was determined by a turbidimetric method (ADVIA 2400 Siemens Healthcare Diagnostics). Laboratory staff was blinded for underlying diseases, HIV status and patient background.

We used cystatin C as a reference to detect a serum creatinine based equation closest to cystatin C to allow reliable future creatinine based GFR estimation in resource limited settings. Creatinine-based eGFR was calculated using the Cockcroft-Gault, [18] the MDRD-4 [19,31] and the CKD-EPI [21] equations. Cystatin C based eGFR was calculated using the formula by van Deventer et al. [32] developed in a comparable cohort in South Africa and verified by the CKD-EPI equations for cystatin C. [33] Table 1 gives lists all formulae used. The accordance of the GFR estimated by creatinine formulae with the cystatin C based values was checked using Bland-Altman-Plots.

**Table 1:** Evaluations used for estimation of GFR.

**Creatinine-based equations:**		
**Cockcroft-Gault:** [18] eGFR = (140-age) x mass [in kg] x 0.85 [if female] / 72 x SCr		
**MDRD-4:** [31] eGFR = 175 x SCr^-1.154^ x age^-0.203^ x 1.212 [if black] x 0.742 [if female]		
**CKD-EPI:** [21]		
**Black American**		
Female	≤0.7 mg/dL:	eGFR = 166 x (SCr/0.7)^-0.329^ x (0.993)^age^
	>0.7 mg/dL:	eGFR = 166 x (SCr/0.7) ^-1.209^ x (0.993)^age^
Male	≤0.9 mg/dL:	eGFR = 163 x (SCr/0.9) ^-0.411^ x (0.993)^age^
	>0.9 mg/dL:	eGFR = 163 x (SCr/0.9) ^-1.209^ x (0.993)^age^
**White or other**		
Female	≤0.7 mg/dL:	eGFR = 144 x (SCr/0.7) ^-0.329^ x (0.993)^age^
	>0.7 mg/dL:	eGFR = 144 x (SCr/0.7) ^-1.209^ x (0.993)^age^
Male	≤0.9 mg/dL:	eGFR = 141 x (SCr/0.9) ^-0.411^ x (0.993)^age^
	>0.9 mg/dL:	eGFR = 141 x (SCr/0.9) ^-1.209^ x (0.993)^age^
**Cystatin C based equations:**		
**Van Deventer:** [32] eGFR = 10^2.35^ x 10^(SCysC [mg/L] x -0.33)^ x 10^(-0.003 x age)^		
**CKD-EPI:** [33]		
**Without correction factors:** eGFR = 76.7 x SCysC^-1.19^		
**With correction factors:** eGFR = 127.7 x SCysC^-1.17^ x age^-0.13^ x 0.91 [if female] x 1.06 [if black]		

Creatinine was measured in mg/dl and IDMS traceable, cystatin C was measured by a turbidimetric method in mg/l; weight measured in kg, age measured in years. Abbreviations: SCr = serum creatinine, SCysC = serum cystatin C

In a first step we compared each creatinine based eGFR to the eGFR derived from cystatin C (formula according to van Deventer), and their performance regarding HIV status. In a second step we compared the performance of the creatinine based eGFR formulae among each other, with and without considering the factor for black Americans.

### Statistical analysis

Data analysis was done in STATA 10 and SAS 9.3. Significance in the differences between HIV-positive and—negative cohorts were tested using appropriate tests according to the underlying distribution. Agreement between different measurement techniques was assessed by Bland-Altman-Plots. [34–36] Bland-Altman graphs were created by plotting the means (x-axes) of two GFR estimation methods against their differences (y-axes). We applied linear regression models to obtain the mean differences and the limits of agreement in presence of heteroscedasticity, as suggested in [35]. In presence of strong non-linearity and differences in the distribution shapes we used quantile regression. [37–39] Plots were grouped according to HIV status. In case of visible differences between HIV- negatives and-positives the mean differences and the limits of agreement were presented separately. Additional analyses were performed to assess the formulae by calculating the absolute and relative bias, the precision, and the accuracy. Firstly, to determine the central distance between two formulae we calculated the overall mean differences (absolute bias) between the estimated GFRs to be compared. The reference eGFR was interpreted to be overestimated by the predicting eGFR if values were < 0, and underestimated if values were > 0. Secondly, we calculated the precision, which is the standard error of the mean differences. Thirdly, we calculated the relative bias which is the division of the mean differences between the two estimates by the reference eGFR value. Fourthly, accuracy between two methods was shown by the proportion of values obtained with method A of those estimated by method B within a margin of 10%, and 30% respectively. Finally we assessed the resulting differences in staging of patients to a level of CKD when applying different formulae.

### Ethics Statement

Participants provided consent in written form or by fingerprint. The study protocol and the consent procedure received ethical clearance by the ethical committee of Heidelberg University and the National Health Sciences Research Committee of Malawi.

## Results

Out of 381 clients approached to participate in the study, 366 consented (95%) and data from 363 participants (48% female) were included in the final analysis. Reasons for the exclusion of three participants were previous ART and two missing samples.

116 (32%) participants were HIV-positive and 247 were HIV-negative (68%) (details see Table 2). Mean BMI between HIV-positives and-negatives differed significantly. Three had previously been diagnosed with kidney disease: two could not specify the disease, the other one reported glomerulonephritis.

**Table 2:** Characteristics of included individuals.

		Overall: Median (IQR) or n (% of total)	HIV+ (% in each category)	HIV—(% in each category)	p-value
**Age**		31 (26–39)	32 (27–37.5)	31 (25–41)	0.81^a^
**Sex**	women	174 (48%)	57 (49%)	117 (47%)	0.75^b^
	men	189 (52%)	59 (51%)	130 (53%)	
**BMI**		22.0 (20.2–24.8)	20.8 (19.0–22.9)	22.6 (20.9–26.2)	<0.001^a^
**History of diabetes mellitus**	Earlier diagnosed	15 (4%)	0 (0%)	16 (6.5%)	0.005^b^
	newly diagnosed	1 (0.03%)			
**History of tuberculosis**		17 (5%)	6 (5%)	11 (4%)	0.835^b^
**History of kidney disease in the past**		3 (1%)	0 (0%)	3 (1%)	0.565^b^
**BP > 140 systolic or > 90 diastolic**		49 (14%)	9 (8%)	40 (16%)	0.028^b^
**Serum creatinine**		0.73 mg/dl (0.63–0.85)	0.69 mg/dl (0.59–0.83)	0.74 mg/dl (0.64–0.85)	0.21^a^
**Serum cystatin C**		0.78 mg/l (0.7–0.89)	0.87 mg/l (0.78–0.98)	0.75 mg/l (0.67–0.84)	<0.001^a^
**CKD stage 3+**	CKD-EPI	7 (1.9%)	2 (1.7%)	5 (2.0%)	0.85^b^
	Cystatin C	11 (3.0%)	5 (4.3%)	6 (2.4%)	0.33^b^

^a^ t-test

^b^ χ^2^-test

The performance of the various GFR estimation formulae was assessed by comparing the different creatinine based equations with the cystatin C based equation by van Deventer et al. [32] in Bland Altman plots. [40]

**Fig 1:**
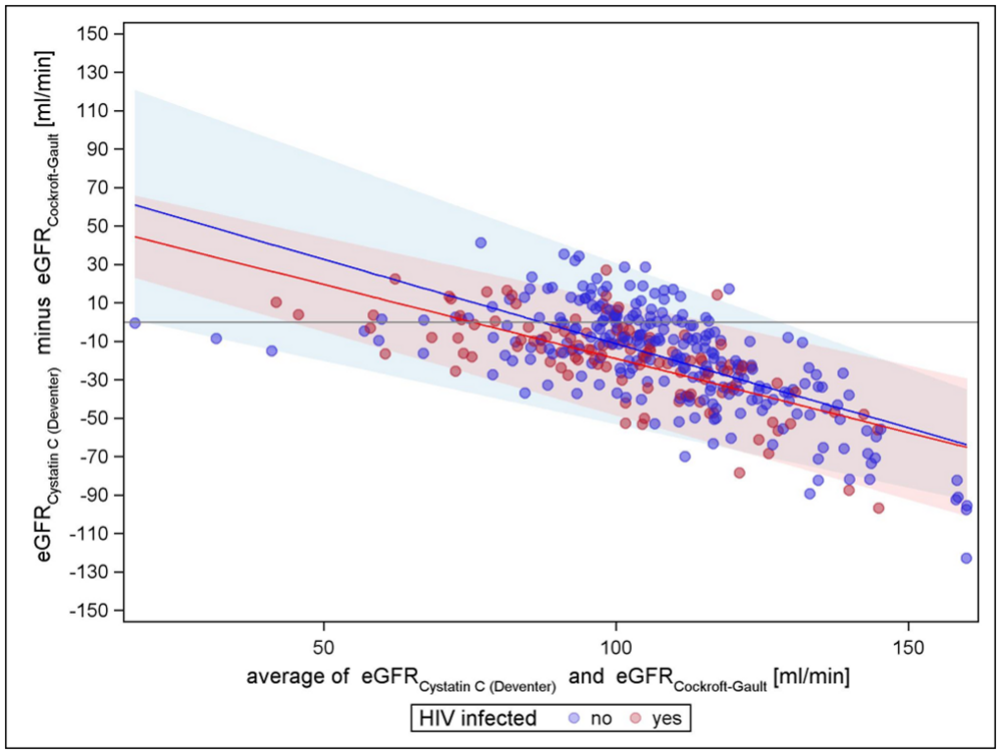
Cystatin C (van Deventer) vs. Cockcroft-Gault. The coloured lines represent the mean differences of the two equations to be compared at every point of the mean of the estimated GFRs, by HIV status; the coloured shaded areas mark the limits of agreement, which are mean- differences plus or minus two standard-deviations. Assuming a normal distribution, 95% of the dots are expected to appear within the limits of agreement. [40] Closer margins reflect a higher agreement of the different methods.

**Fig 2:**
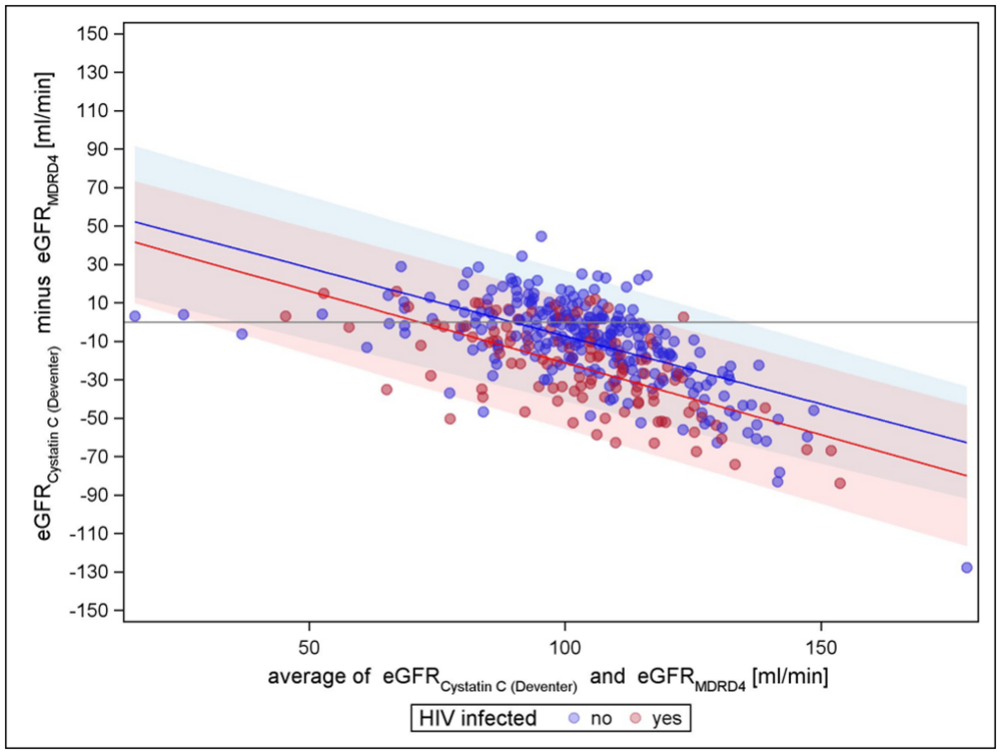
Cystatin C (van Deventer) vs. MDRD-4 (without factor for black Americans).

**Fig 3:**
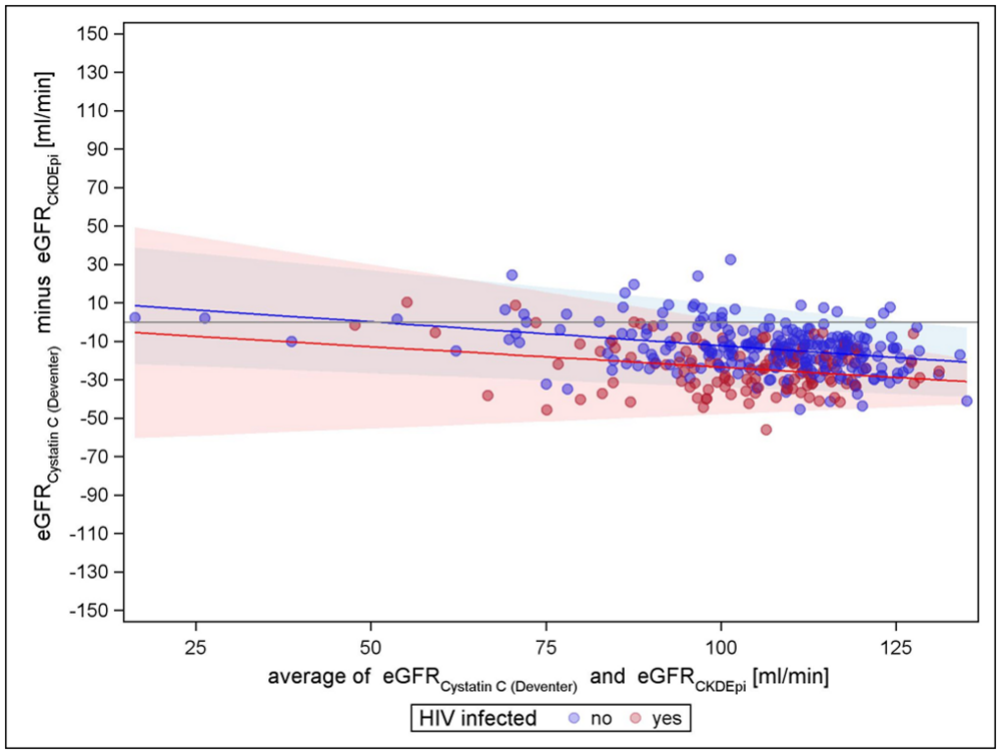
Cystatin C (van Deventer) vs. CKD-EPI (without factor for black Americans).

Figs 1–3 present the comparisons of the different creatinine equations with the cystatin C equation (van Deventer) without the correction factor for black Americans. All comparisons show an increasing agreement with increasing mean eGFR for HIV negatives (narrowing limits of agreement). All comparisons show differences with respect to HIV status; hence, the creatinine based eGFR of HIV positives seems to be higher in general. For cystatin C vs. CKD-EPI, the mean differences regression line remains closest to zero over the entire range for HIV-negatives, indicating almost no trend in the bias, while for cystatin C vs. MDRD-4 and cystatin C vs. Cockcroft-Gault, the course of the regression line has a strong tendency. The Cockcroft-Gault formula shows the smallest mean differences between HIV-positives and-negatives, most likely because it controls for body weight. Altogether, cystatin C vs. CKD-EPI is least biased for both HIV negatives and positives and has the tightest limits of agreement. A sensitivity analysis using the cystatin C based equation evaluated by CKD-EPI showed similar results as the Cystatin C formula by Deventer et al., CKD-EPI creatinine still performed best compared to Cockcroft-Gault and MDRD-4 (see supplementary figures in S1, S2, S3, S4, S5, and S6 Figs). However, the variation was increased in general.

**Fig 4:**
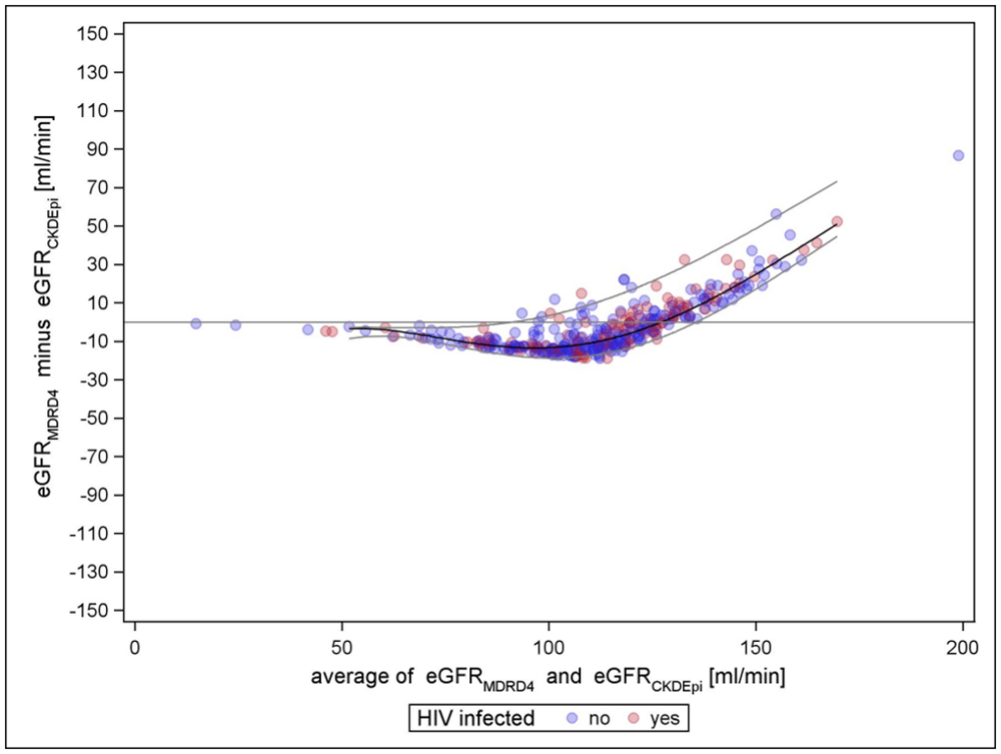
MDRD-4 (without factor for black Americans) vs. CKD-EPI (without factor for black Americans).

Directly comparing the CKD-EPI and the MDRD-4 formula without the factor for black Americans shows completely different performance at higher mean eGFR, although values at lower levels are rather similar. Further, a difference between HIV-positives and-negatives cannot be recognised here (Fig 4).

**Fig 5:**
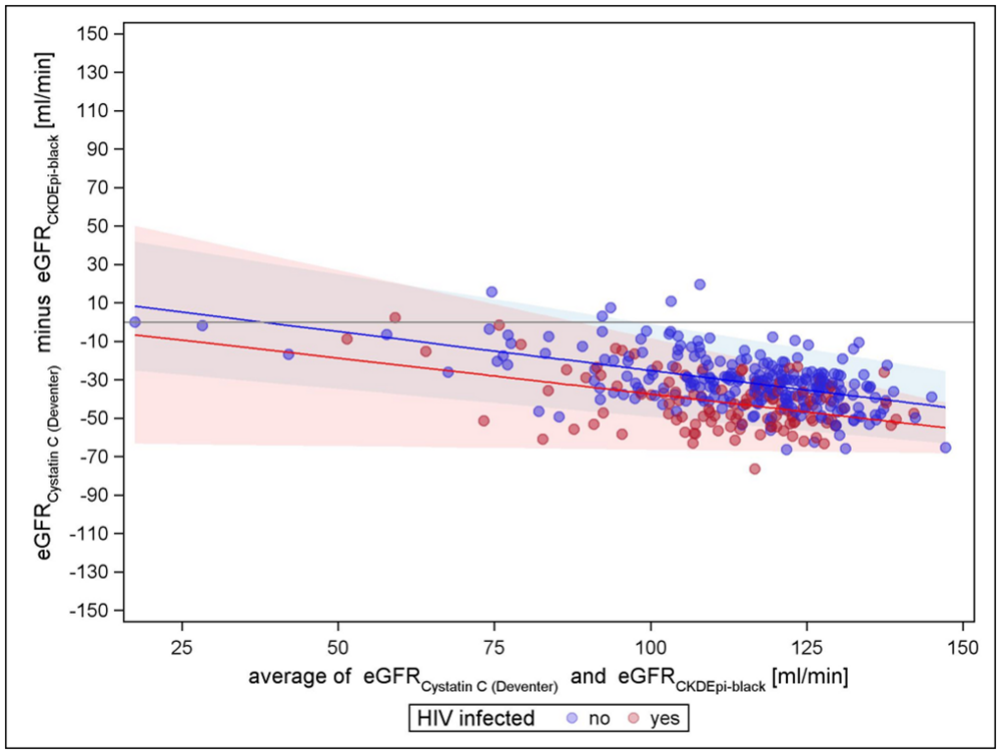
Cystatin C (van Deventer) versus MDRD4 with factor for black Americans.

**Fig 6:**
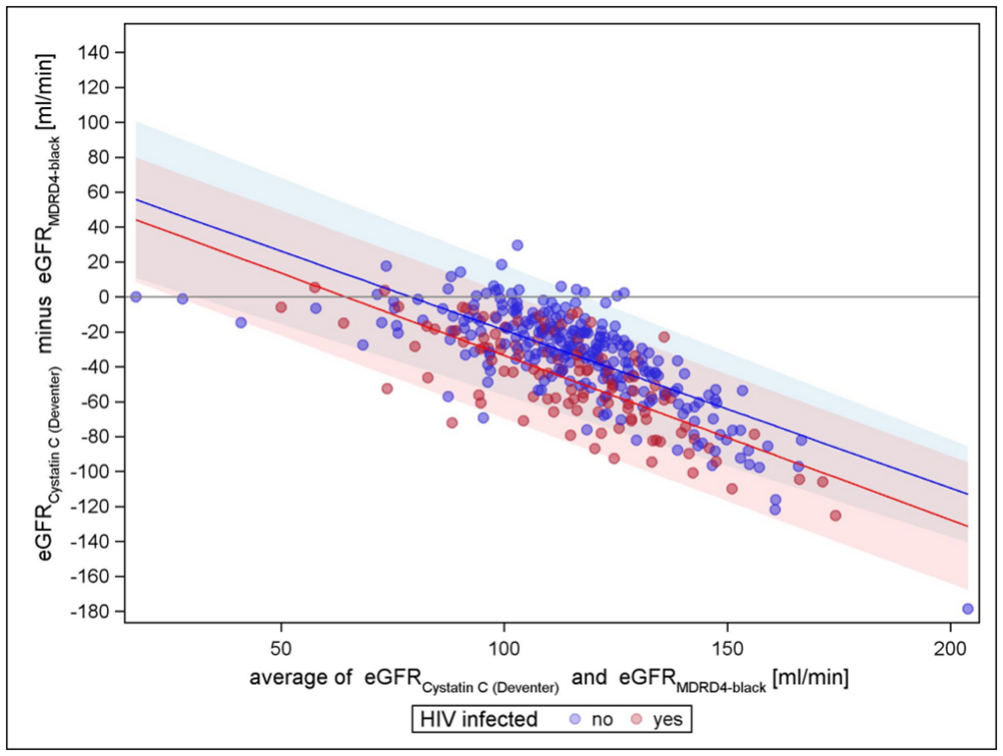
Cystatin C (van Deventer) versus CKD-EPI with factor for black Americans.

Applying the factor for black Americans in the creatinine based formulae yielded a similar distribution pattern compared to the plot of CKD-EPI without the factor for black Americans, but witha stronger bias tendency. For MDRD-4 with factor for black Americans the mean also shifted towards lower values, mostly < 0, compared to MDRD-4 without this adjustment factor (Figs 5 and 6).

**Fig 7:**
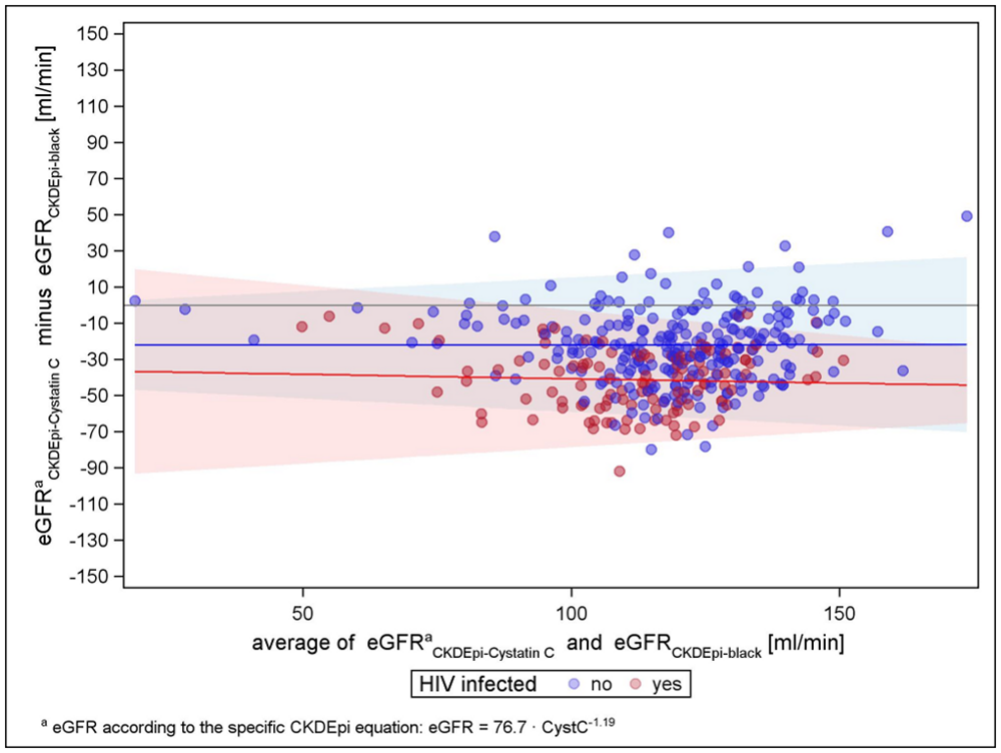
CKD-EPI-Cystatin-C versus CKD-EPI with factor for black Americans.

**Fig 8:**
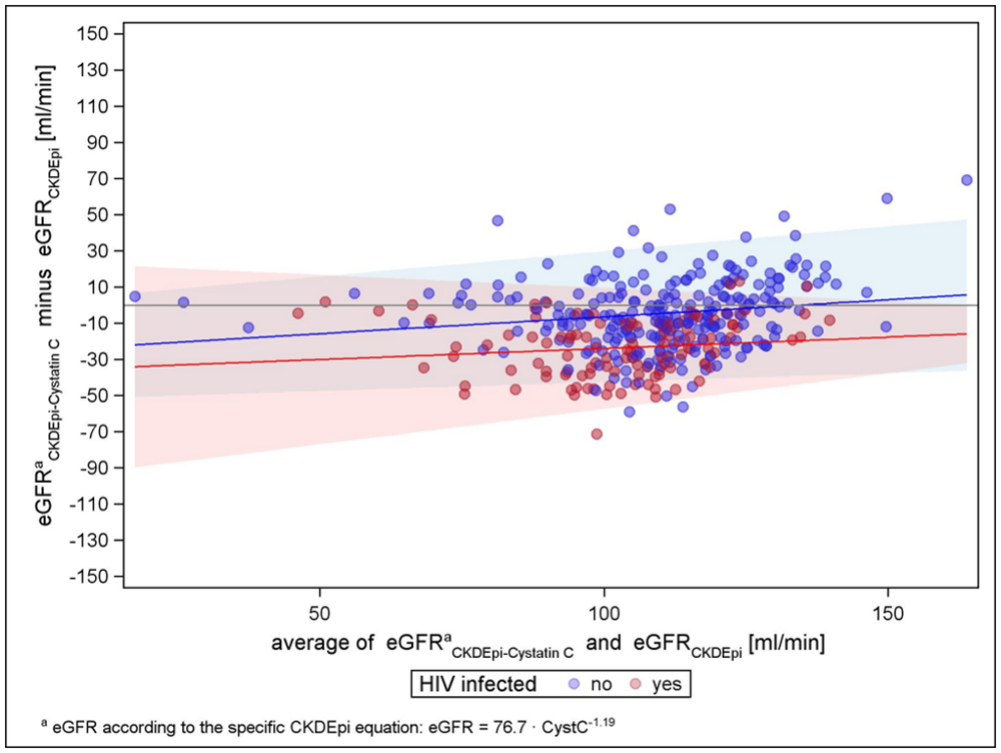
CKD-EPI-Cystatin-C versus CKD-EPI without factor for black Americans.

To explore whether the difference between formulae with and without factor for black Americans was confounded by the cystatin C equation calculated by van Deventer et al. in black South-Africans, we compared CKD-EPI with and without the factor with the CKD-EPI cystatin C equation by Stevens et al., which has been developed based on results of different pooled cohorts with GFR measured by iothalamate. [20,33] Both figures show similar distribution pattern, with mean differences shifted towards lower numbers for both HIV-negatives and-positives in case the factor is considered (see Figs 7 and 8).

By numerical assessment CKD-EPI, Cockcroft-Gault and MDRD-4 showed a similar overall absolute bias with a larger bias for HIV-positives (Table 3). Comparing Cockcroft-Gault with MDRD-4 or CKD-EPI, respectively, the overall bias was small but of different direction in HIV-positives and-negatives. Comparing against formulae with factor for black Americans yielded the worst bias.

**Table 3:** Absolute bias (mean differences) and precision (standard error of the mean differences), and relative bias of two formulas to be compared.

*absolute bias (precision)*, *relative bias*	Overall (N = 363)	HIV- (N = 247)	HIV+ (N = 116)
Cystatin C vs. CKD-EPI	-17.0 (0.7), 17.6%	-13.7 (0.8), 13.8%	-24.1 (1.1), 26.5%
Cystatin C vs. MDRD4	-14.3 (1.2), 14.8%	-10.1 (1.4), 10.3%	-23.1 (2.1), 25.5%
Cystatin C vs. Cockcroft-Gault	-18.7 (1.3), 19.4%	-18.4 (1.7), 18.6%	-19.4 (2.1), 21.4%
Cystatin C vs. CKD-EPI modified*	-34.5 (0.8), 35.8%	-31.1 (0.9), 31.4%	-41.8 (1.3), 46.0%
Cystatin C vs. MDRD4 modified*	-37.5 (1.5), 39.0%	-33.1 (1.7), 33.4%	-47.1 (2.6), 51.8%
CKD-EPI cystatin C^a^ vs. CKD-EPI modified*	-28.1 (1.1), 27.3%	-22.0 (1.3), 20.3%	-41.3 (1.6), 45.2%
CKD-EPI cystatin C^a^ vs. CKD-EPI	-10.6 (1.1), 10.3%	-4.5 (1.3), 4.2%	-23.5 (1.5), 25.7%
Cockcroft-Gault vs. MDRD4	4.4 (1.3), -3.8%	8.2 (1.6), -7.0%	-3.7 (2.0), -3.4%
Cockcroft-Gault vs. CKD-EPI	1.7 (1.2), -1.5%	4.7 (1.5), -4.0%	-4.6 (1.8), 4.2%
MDRD4 vs. CKD-EPI	-2.7 (0.7), 2.4%	-3.5 (0.9), 3.2%	-0.9 (1.3), 0.8%

* with factor for black Americans

^a^ CKD-EPI equation: eGFR = 76.7 x CystC^-1.19^

Referencing to cystatin C, the precision (standard error of the mean) showed best results for CKD-EPI (see Table 3); higher values for MDRD-4 and Cockcroft-Gault indicate greater variability of those methods in comparison to cystatin C, even though the absolute bias slightly differs. The magnitude of the relative bias was always close to the absolute bias. The supplementary table in S1 Table shows the results when referencing to one of the CKD-EPI cystatin C equations. Again, CKD-EPI shows the lowest variability.

We counted all GFR values obtained with formula A lying within a range of plus/minus 30% of the corresponding value of formula B. Accuracy with reference to cystatin C was highest for CKD-EPI (Table 4). However, differences between the HIV groups of HIV-negative and-positive were large. Best of all performed MDRD-4 versus CKD-EPI. Applying the factor for black Americans resulted in lower accuracy. Limiting the agreement range to 10% drastically decreased accuracy (see Table 4 and supplementary material in S2 Table).

**Table 4:** 30% and 10% accuracy.

	30% accuracy			10% accuracy		
*%of estimates A within %range of B*	all	HIV-	HIV+	all	HIV-	HIV+
CKD-EPI vs. cystatin C	81%	92%	59%	24%	29%	12%
MDRD4 vs. cystatin C	76%	83%	60%	33%	38%	22%
Cockcroft-Gault vs. cystatin C	69%	69%	71%	28%	31%	21%
MDRD4 vs. CKD-EPI	99%	99%	99%	51%	49%	53%
Cystatin C vs. CKD-EPI modified*	67%	78%	43%	6%	8%	2%

* with factor for black Americans

The resulting differences in classification agreement of CKD stages when comparing two formulae are shown in Table 5 (see additional material in S3 Table), using stage 3 respectively stage 2 as cut off. Regarding stage 3 and above, creatinine based CKD-EPI yields a prevalence of 1.9% (7 cases; not visible in the table), and cystatin C (van Deventer) of 3% (11 cases), for instance. However, altogether 8 cases were classified differentially by both methods. With CKD stage 2 as cut off, CKD-EPI yields a prevalence of 9.6% (35 cases; not visible in the table), and cystatin C a prevalence of 27.8% (101 cases), with around 20% of the persons classified stage 2 or higher by cystatin C but not by CKD-EPI at the same time.

**Table 5:** Discrepancies in staging results, cut off stage 3 and 2.

Discrepant results CKD stage 3 A versus B	**Same CKD stages splitting ≥ 3 and < 3 (%)**	**CKD stage ≥ 3: A, not B (%)**	**CKD stage ≥ 3: B, not A (%)**
CKD-EPI vs. cystatin C	355 (97.8)	6 (1.7)	2 (5.5)
MDRD4 vs. cystatin C	354 (97.5)	4 (1.1)	5 (1.4)
Cockcroft-Gault vs. cystatin C	355 (97.8)	4 (1.1)	4 (1.1)
CKD-EPI modified* vs. cystatin C	355 (97.8)	1 (0.3)	7 (1.9)
*Discrepant results CKD stage 2 A versus B*	**Same CKD stages splitting ≥ 2 and < 2 (%)**	**CKD stage ≥ 2: A, not B (%)**	**CKD stage ≥ 2: B, not A (%)**
CKD-EPI vs cystatin C	285 (78.5)	6 (1.7)	72 (19.8)
MDRD4 vs cystatin C	285 (78.5)	23 (6.3)	55 (15.2)
Cockcroft-Gault vs cystatin C	281 (77.4)	22 (6.1)	60 (16.5)
CKD-EPI modified* vs. cystatin C	279 (76.9)	1 (0.3)	83 (22.)

* with factor for black Americans

## Discussion

In this study we validate different creatinine based equations for GFR in 363 Malawian adults, comprising HIV-negative and-positive individuals, in comparison with the cystatin C based equation (van Deventer). It further highlights the eGFR differences in HIV-positive and-negative individuals in a SSA-country and scrutinizes the use of a correction factor designed for black Americans. The CKD-EPI creatinine based formula turned out to currently best assess eGFR in our setting, although the obtained CKD classification results still entail uncertainties. When referencing to any CKD-EPI cystatin C equation in a sensitivity analysis, CKD-EPI creatinine also performed best. The validation showed considerable differences in performance/accuracy of the equations depending on the HIV status. In HIV-positives CKD-EPI eGFR values are systematically overestimated in relation to cystatin C. Introducing the adjustment factor for black Americans in the creatinine based formulae further overestimates GFR. Despite some remaining uncertainties, we therefore recommend using the creatinine based CKD-EPI formula without the factor for black Americans, in SSA contexts. Further research should investigate the reasons behind the differences in HIV-negatives and-positives and identify adjustment variables such as BMI.

With regard to HIV-positive individuals the importance of renal dysfunction and HIV related morbidity and mortality has been highlighted in the EuroSIDA cohort and will become increasingly important for HIV patients in Africa, where renal impairment is not routinely diagnosed. [41]

GFR estimation is essential to assess the burden of CKD in a population. Data from Malawi itself is scarce: There is only one study with 526 ART-naïve HIV-positives using the Cockcroft-Gault-formula to investigate severe renal impairment. [42] In contrast, our study for the first time systematically assesses the performance of different eGFR formulae and also includes a group of HIV-negative Malawian adults, even though it may not be a representative sample of the general adult Malawian population.

### Comparison of creatinine based equations to cystatin C estimated GFR

The CKD-EPI-formula showed the best performance at all levels of eGFR compared to the cystatin C (van Deventer) reference values in HIV-negatives, with acceptable limits of agreement, and almost no tendency regarding the bias of the mean differences of the eGFRs. However, differences in the bias result in different CKD stage classifications, which is especially important in the transition from CKD stage 2 to 3. Considering mean eGFR values lower 60, which corresponds to CKD stage 3 and higher, MDRD-4 and Cockcroft-Gault visually seem to overestimate CKD in comparison to cystatin C, whereas at higher eGFR both formulae underestimate CKD. This is consistent with the discrepant classification results for CKD stage 2 and higher. These results concur with studies that found the CKD-EPI equation more accurate than the MDRD-4-equation overall and across most subgroups especially in eGFR-levels > 60 ml/min/1.73m². [43,44]

The overall absolute bias of CKD-EPI versus cystatin C was similar compared to MDRD-4. However, the absolute bias is only an overall measure, not considering any trends or differences in various eGFR categories and levels. CKD-EPI precision against cystatin C was closest to zero, compared to MDRD-4 and Cockcroft-Gault and therefore suggesting a better fit. In addition, 30% accuracy of CKD-EPI to Cystatin C was relatively high for HIV-negatives. In summary, although established in a cohort living in a high-income country with different conditions of life, and a low prevalence of impaired renal function, the creatinine based CKD-EPI formula seems to yield results closest to the assumed GFR values, represented here by cystatin C (van Deventer). The fact that the level of agreement remained similar between CKD-EPI and a second cystatin C formula (CKD-EPI cystatin C) underpins the statement that the creatinine based CKD-EPI formula without the factor for black Americans is a useful initial marker to estimate GFR in HIV-negatives, as suggested by others. [26,45]

The clinically relevant classification into CKD stages ≥ 3 differs, depending on which formula is applied, but only in a few cases as the overall number of CKD cases was small. Hence, the same calculation was repeated with stage 2 as cut off point. Here, cystatin C is stricter in classifying CKD stages, compared to the others, which is in line with the on average higher eGFR values achieved by CKD-EPI. Following the Bland-Altman plots, it can be assumed, that a large portion of cases classified as CKD by cystatin C but not by CKD-EPI are HIV positives.

The results of other studies which showed that the CKD-EPI equation classified fewer individuals having CKD and better categorized mortality-risks and end stage renal disease (ESRD) probability than the MDRD-equation back these findings. [46]

### Different performances in GFR estimation regarding HIV status

In our study the distribution of age and sex were comparable between HIV-positives and negatives and therefore age should not influence any differences between the groups. However, there was a significant and expected difference in BMI between HIV positive and HIV negative participants. For CKD-EPI the mean differences regression line of HIV-positives is always below zero, indicating constant overestimation of creatinine based eGFR. For all other equations the mean differences regression lines depend on the level of eGFR. However, the smallest distance between the mean differences regression lines of the HIV-positives and-negatives is observable between cystatin C based equations and Cockcroft-Gault. This finding results most likely on the one hand from cystatin C being almost independent of body weight and on the other hand from the fact that Cockcroft-Gault is the only creatinine based formula considering body weight, and weight was significantly different between HIV-positives and-negatives. This is further supported by median serum creatinine and cystatin C. Serum creatinine was lower in HIV-positives compared to-negatives, which is consistent with lower BMI, but median serum cystatin C was significantly higher in HIV-positives. Therefore, cystatin C seems to indicate a real difference between HIV-positives and-negatives, independent of BMI, therefore directly describing differences in kidney function. In consequence, applying creatinine based eGFR formulae in HIV-positives without adjusting for BMI (or other related confounders) tends to overestimate GFR and as a result underestimate CKD burden in this specific group. However, since muscle mass is the important factor which influences creatinine, this could be a specific problem when first diagnosing HIV or in end-stage HIV disease, as muscle mass may increase substantially under antiretroviral treatment otherwise. This observation may also be relevant for conditions other than HIV associated with low weight/BMI.

### Agreement between creatinine based equations

Comparing the creatinine based formulae with each other the Bland Altman plot of MDRD-4 versus CKD-EPI is most conspicuous. Although both formulae are close in terms of absolute bias and the precision is high (indicated by a low value), they show completely different behavior in eGFR mean values above 90. The same pattern has been observed by other studies [47] In moderate and severe CKD cases the MDRD-4 formula is more accurate than Cockcroft-Gault, but it tends to underestimate kidney function in individuals with eGFR > 90 ml/min/1.73m³ and therefore to over-diagnose CKD. [48] This issue has been addressed with the development of the CKD-EPI formula in 2009 which remedies this over-diagnosis and keeps the same accuracy in eGFR < 90 ml/min/1.73 m². [21] Regardless of the recent formula development, the Cockcroft-Gault formula, introduced already in 1976, is still used quite often, although it measures creatinine clearance and does not consider the tubular secretion, hence overestimates GFR in general. [49] From a clinical perspective despite these differences regarding prediction of clinical outcomes the Cockcroft Gault and CKD-EPI formula worked equally well in the predominantly male Euro SIDA Cohort.[50]

### Application of Black American correction factors

Considering the factor for black Americans in MDRD-4 and CKD-EPI resulted in higher estimated GFR-levels in general, validated by cystatin C. This might be due to the fact, that Malawian people have a different diet intake and way of living compared to most black Americans living in the global north. The serum creatinine levels of black Americans seem to be generally higher than those of white American people or other ethnic groups in the US. [51,52] As we know serum creatinine levels vary with stress, hypertension etc. which might possibly be confounded by stress linked to direct and indirect racial pressure in the USA, [53] however, also epi-genetic selection among black Americans may play a role. [23–25] Our findings suggest that this correction factor should not be used for Malawians.

This is supported by other studies which found that using eGFR formulae with the factor for black Americans leads to an overestimation of measured GFR in South Africa, [54] as well as in Ghana. [26] Delanaye et al. stated in their review, based on multiple findings, that although the ethnic factor leads overall to accurately estimated GFR in black Americans, it does not seem to be applicable in African populations. [55]

### Limitations of the study

Due to the cross-sectional character of our study we obtained samples for creatinine only once. We were unable to conduct a true gold-standard investigation. Therefore, we chose cystatin C as a reference since it is less dependent on physiological parameters. However, cystatin C has its own limitations and also imperfectly represents the unknown real GFR. [27] Gold-standard measuring of GFR by inulin- or iohexol-clearance, was not possible in the outpatient and resource-constrained study setting. Using cystatin C appeared to be an acceptable alternative. We acknowledge that we did not use the certified reference material for cystatin C that has been developed by the International Federation of Clinical Chemistry and Laboratory Medicine (IFCC) as recommended by KDIGO in 2012. [20] This had not yet been introduced at the university laboratory performing the analysis, however, all testsfulfilled highest quality control standards.

Our study population is not representative of the adult general population in Malawi. Since the study participants were enrolled from a HIV testing centre we cannot preclude selection bias in the HIV-negative group as people who were tested HIV-negative also may have been sicker than the general population. These differences in population characteristics could have additionally confounded serum creatinine, resulting in biased eGFR in the HIV-negatives. Furthermore, the prevalence of CKD was relatively low in our study population, allowing inference mainly at relative high eGFR values. However, the aim of this study was not to estimate the prevalence of CKD but to assess the performance of the different equations for eGFR which should be less influenced by this selection.

## Conclusions and Recommendations

We suggest applying the creatinine based CKD-EPI-formula without the factor for black Americans to estimate the renal function in HIV-negative Malawian people and other similar cohorts in SSA. We recommend caution when applying this formula in HIV-positive individuals, because eGFR levels are most probably overestimated. We recommend any study taking renal function and HIV status into account to use cystatin C based equations for the HIV-positive individuals.

Since the sensitivity of creatinine-based formulae in general is low [56] especially in the important transition from CKD stage two to three due to the hyperbolic association between creatinine clearance and plasma creatinine, we suggest establishing a two-step testing approach, if possible. Subjects classified in the transition stages two and three based on creatinine should be assessed a second time according to their cystatin C levels, especially if their HIV-status is positive. This advanced testing approach should drastically reduce misclassification, but only slightly increase processing costs.

Cystatin C measurement is not yet standard practice in Malawi and many other countries in SSA because the costs are considerably higher compared to creatinine measurements. If this remains the case, the application of a creatinine based CKD-EPI formula which corrects for confounders such as HIV status and BMI should be aspired to. However, since muscle mass is the important influence factor on serum creatinine, and clearly relates to BMI in underweight individuals in absence of body fat only, the inclusion of BMI has to be done carefully. In the long run, we highly recommend to foster the application of cystatin C based eGFR as a common standard to more accurately assess individual kidney function.

## Supporting Information

S1 Dataset Complete dataset, anonymized. Data of all study participants in Malawi, containing individual characteristics, blood pressure, specific diagnosis, serum creatinine and cystatin C values, and calculated eGFR according to different formulae.
(XLS)

S1 Fig CKD-EPI-Cystatin-C versus Cockcroft-Gault. CKD-EPI equation: eGFR = 76.7 x CystC^-1.19^
(TIF)

S2 Fig CKD-EPI-Cystatin-C versus MDRD-4. CKD-EPI equation: eGFR = 76.7 x CystC^-1.19^
(TIF)

S3 Fig CKD-EPI-Cystatin-C versus CKD-EPI (without factor for black Americans). CKD-EPI equation: eGFR = 76.7 x CystC^-1.19^
(TIF)

S4 Fig CKD-EPI-Cystatin-C versus Cockcroft-Gault. CKD-EPI equation: eGFR = 127.7 x CystC^-1.17^ x age^-0.13^ x 0.91_[if female]_ x 1.06_[if black]_
(TIF)

S5 Fig CKD-EPI-Cystatin-C versus MDRD-4. CKD-EPI equation: eGFR = 127.7 x CystC^-1.17^ x age^-0.13^ x 0.91_[if female]_ x 1.06_[if black]_
(TIF)

S6 Fig CKD-EPI-Cystatin-C versus CKD-EPI (without factor for black Americans). CKD-EPI equation: eGFR = 127.7 x CystC^-1.17^ x age^-0.13^ x 0.91_[if female]_ x 1.06_[if black]_
(TIF)

S1 Table Absolute bias (mean differences) and precision (standard error of the mean differences), and relative bias of two formulas to be compared; further comparisons. (DOC)

S2 Table Method comparison 30% and 10% accuracy, further comparisons 30% and 10% accuracy, further comparisons. (DOC)

S3 Table Method comparison staging results, further comparisons. (DOC)
