# Supplementary figures and images for: Comparison of Various Equations for Estimating GFR in Malawi: How to Determine Renal Function in Resource Limited Settings?
Source: PLoS One. 2015 Jun 17;10(6):e0130453. doi: 10.1371/journal.pone.0130453 (PMC4470826; doi:10.1371/journal.pone.0130453)

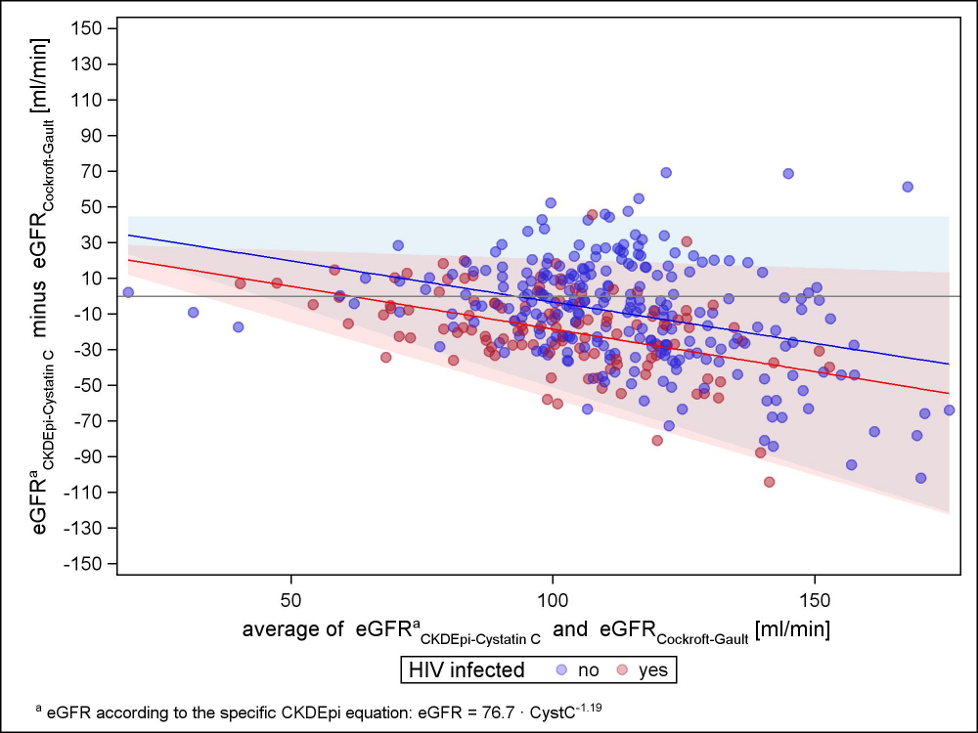

Supplement: S1 Fig — CKD-EPI equation: eGFR = 76.7 x CystC-1.19 (TIF) [file pone.0130453.s002.tif]

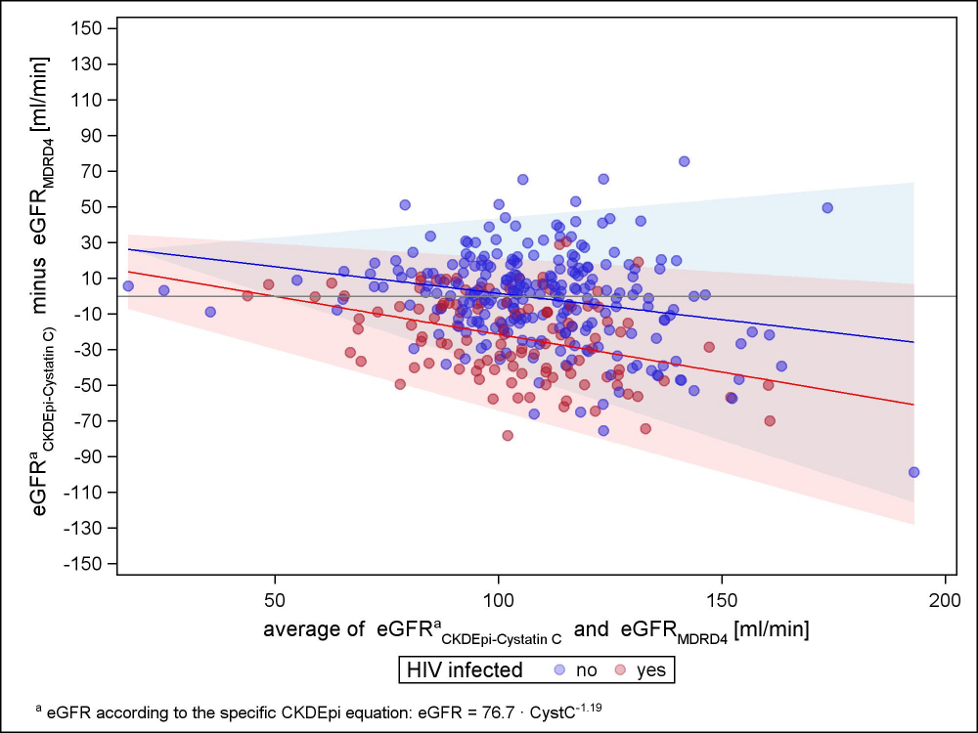

Supplement: S2 Fig — CKD-EPI equation: eGFR = 76.7 x CystC-1.19 (TIF) [file pone.0130453.s003.tif]

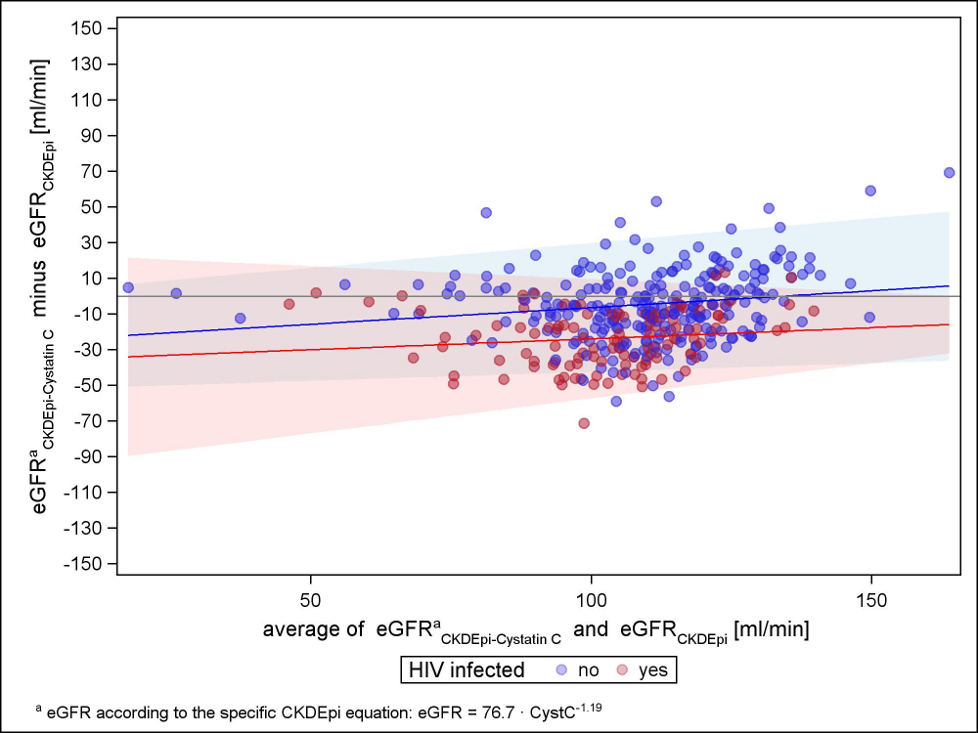

Supplement: S3 Fig — CKD-EPI equation: eGFR = 76.7 x CystC-1.19 (TIF) [file pone.0130453.s004.tif]

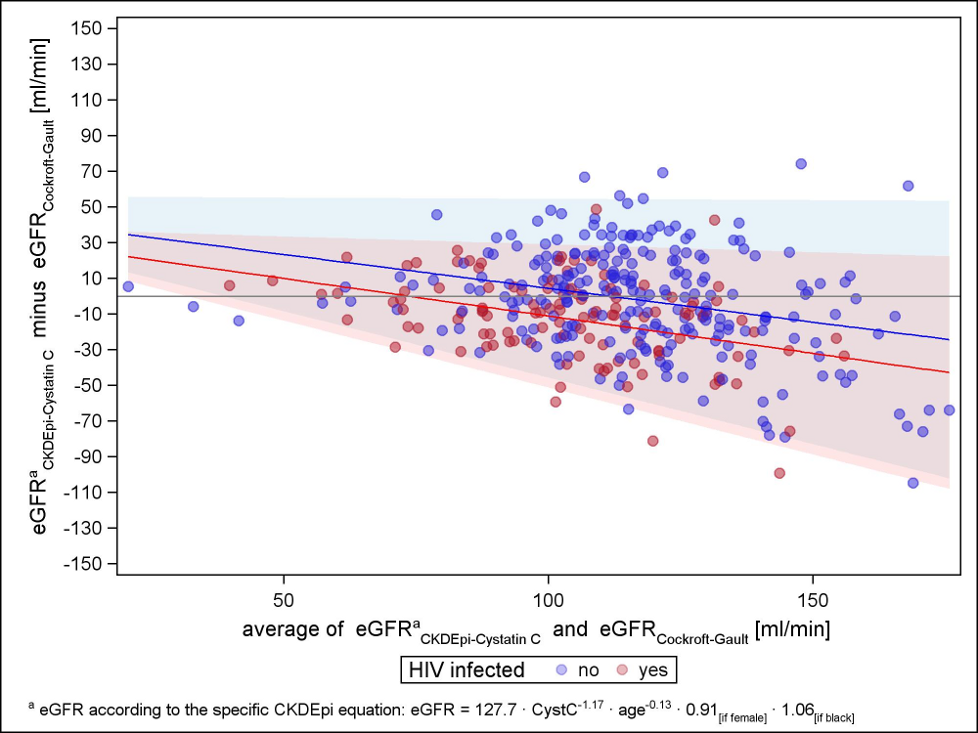

Supplement: S4 Fig — CKD-EPI equation: eGFR = 127.7 x CystC-1.17 x age-0.13 x 0.91[if female] x 1.06[if black] (TIF) [file pone.0130453.s005.tif]

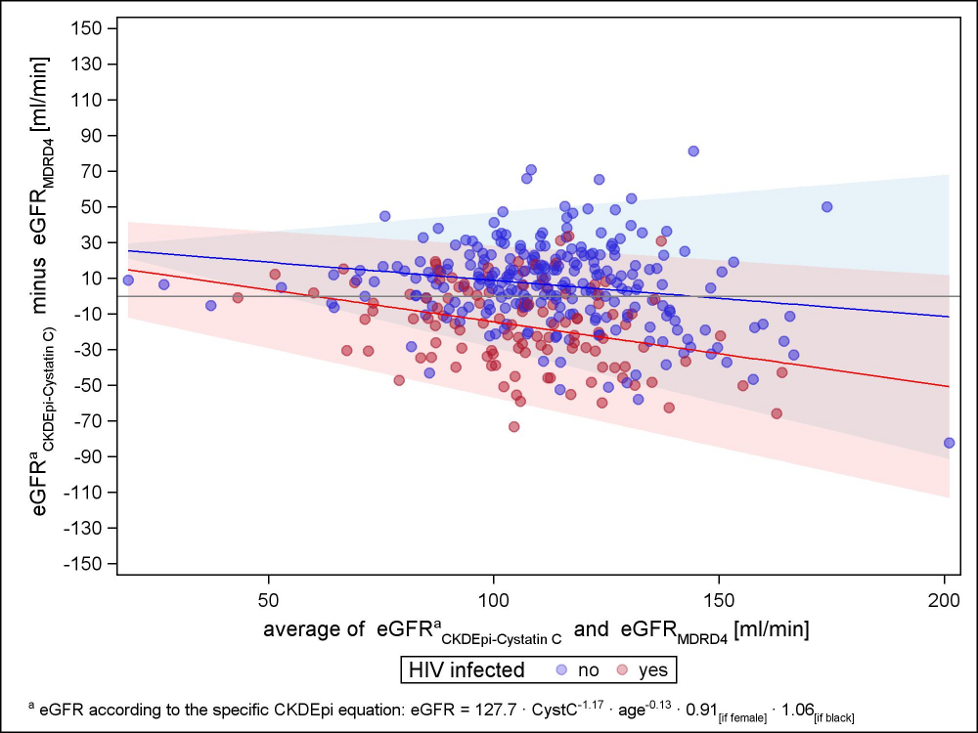

Supplement: S5 Fig — CKD-EPI equation: eGFR = 127.7 x CystC-1.17 x age-0.13 x 0.91[if female] x 1.06[if black] (TIF) [file pone.0130453.s006.tif]

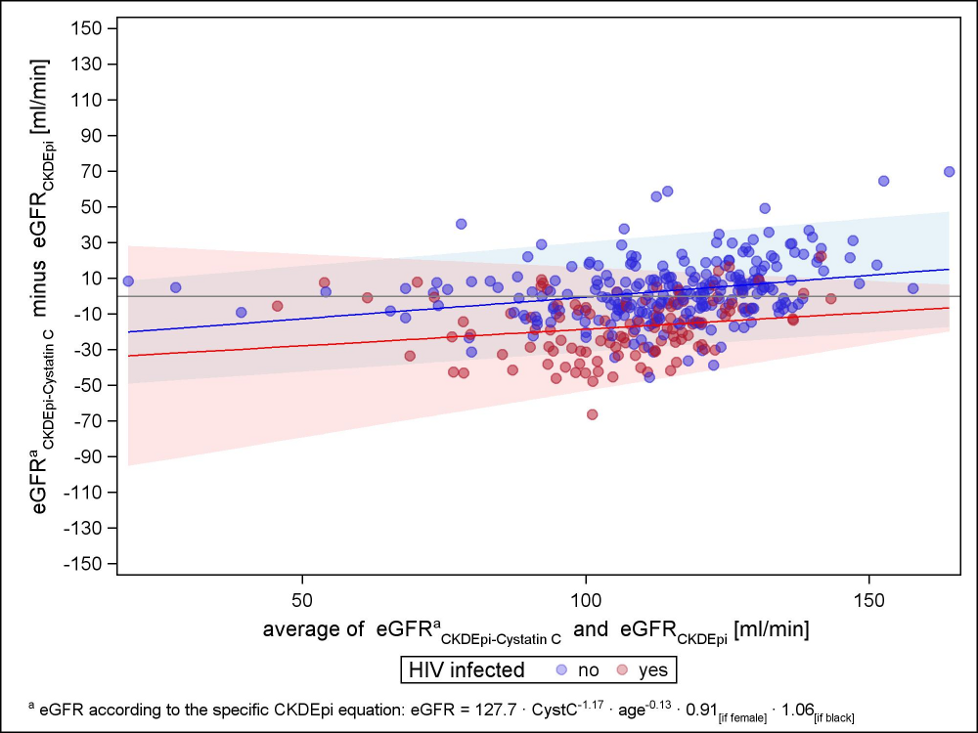

Supplement: S6 Fig — CKD-EPI equation: eGFR = 127.7 x CystC-1.17 x age-0.13 x 0.91[if female] x 1.06[if black] (TIF) [file pone.0130453.s007.tif]
