# Supplementary material for: Comparison of Various Equations for Estimating GFR in Malawi: How to Determine Renal Function in Resource Limited Settings?
Source: PLoS One. 2015 Jun 17;10(6):e0130453. doi: 10.1371/journal.pone.0130453 (PMC4470826; doi:10.1371/journal.pone.0130453)
Supplement: S1 Table — (DOC) [file pone.0130453.s008.doc]

| absolute bias (precision), relative bias | **Overall (N=363)** | **HIV- (N=247)** | **HIV+ (N=116)** |
| --- | --- | --- | --- |
| CKD-EPI cystatin C^b^ vs. CKD-EPI | -3.6 (1.0), 3.3% | 2.8 (1.1), -2.5% | -17.4 (1.5), 17.8% |
| CKD-EPI cystatin C^b^ vs. Cockroft-Gault | -5.4 (1.5), 4.9% | -1.9 (1.9), 1.6% | -12.8 (2.2), 13.1% |
| CKD-EPI cystatin C^b^ vs. MDRD4 | -0.9 (1.3), 0.9% | 6.4 (1.5), -5.5% | -16.5 (2.2), 16.9% |

S1 table: Absolute bias (mean differences) and precision (standard error of the mean differences), and relative bias of two formulas to be compared; further comparisons

^b^ CKD-EPI equation: eGFR = 127.7 x CystC^-1.17^ x age^-0.13^ x 0.91_[if female]_ x 1.06_[if black]_
