# Supplementary material for: Comparison of Various Equations for Estimating GFR in Malawi: How to Determine Renal Function in Resource Limited Settings?
Source: PLoS One. 2015 Jun 17;10(6):e0130453. doi: 10.1371/journal.pone.0130453 (PMC4470826; doi:10.1371/journal.pone.0130453)
Supplement: S2 Table — (DOC) [file pone.0130453.s009.doc]

|  | **30% accuracy** | | | **10% accuracy** | | |
| --- | --- | --- | --- | --- | --- | --- |
| *%of estimates A within %range of B* | **all** | **HIV-** | **HIV+** | **all** | **HIV-** | **HIV+** |
| Cockcroft-Gault vs. MDRD4 | 83% | 80% | 91% | 44% | 44% | 44% |
| Cockcroft-Gault vs. CKD-EPI | 88% | 86% | 92% | 40% | 42% | 37% |
| Cystatin C vs. MDRD4 modified* | 61% | 70% | 41% | 13% | 17% | 7% |
| CKD-EPI cystatin C^a^ vs. CKD-EPI modified* | 72% | 82% | 50% | 19% | 26% | 3% |
| CKD-EPI cystatin C^a^ vs. CKD-EPI | 86% | 92% | 72% | 37% | 43% | 25% |
| CKD-EPI cystatin C^b^ vs. CKD-EPI | 87% | 92% | 72% | 47% | 55% | 31% |
| CKD-EPI cystatin C^b^ vs. Cockroft-Gault | 82% | 94% | 72% | 32% | 31% | 34% |
| CKD-EPI cystatin C^b^ vs. MDRD4 | 78% | 79% | 69% | 32% | 34% | 27% |

Table S2: Method comparison 30% and 10% accuracy, further comparisons

* with factor for black Americans
^a^ CKD-EPI equation: eGFR = 76.7 x CystC-1.19
^b^ CKD-EPI equation: eGFR = 127.7 x CystC-1.17 x age-0.13 x 0.91[if female] x 1.06[if black]
