# Supplementary material for: Comparison of Various Equations for Estimating GFR in Malawi: How to Determine Renal Function in Resource Limited Settings?
Source: PLoS One. 2015 Jun 17;10(6):e0130453. doi: 10.1371/journal.pone.0130453 (PMC4470826; doi:10.1371/journal.pone.0130453)
Supplement: S3 Table — (DOC) [file pone.0130453.s010.doc]

S3 table: Method comparison staging results, further comparisons

| *Discrepant results CKD stage 3 A versus B* | **Same CKD stages splitting ≥ 3 and < 3 (%)** | **CKD stage ≥ 3: A, not B (%)** | **CKD stage ≥ 3: B, not A (%)** |
| --- | --- | --- | --- |
| Cockcroft-Gault vs. MDRD4 | 356 (98.1) | 4 (1.1) | 3 (0.8) |
| CKD-EPI vs. Cockcroft-Gault | 357 (98.3) | 1 (0.3) | 5 (1.4) |
| CKD-EPI vs. MDRD4 | 360 (99.2) | 0 (0.0) | 3 (0.8) |
| Cystatin C vs. MDRD4 modified* | 355 (97.8) | 7 (1.9) | 1 (0.3) |
| CKD-EPI modified* vs. CKD-EPI cystatin C^a^ | 356 (98.1) | 0 (0.0) | 7 (1.9) |
| CKD-EPI vs. CKD-EPI cystatin C^a^ | 356 (98.1) | 1 (0.3) | 6 (1.7) |
| CKD-EPI vs. CKD-EPI cystatin C^b^ | 359 (98.9) | 1 (0.3) | 3 (0.8) |
| Cockroft-Gault vs. CKD-EPI cystatin C^b^ | 357 (98.3) | 4 (1.1) | 2 (0.6) |
| MDRD4 vs. CKD-EPI cystatin C^b^ | 256 (70.1) | 4 (1.1) | 3 (0.8) |
| *Discrepant results CKD stage 2 A versus B* | **Same CKD stages splitting ≥ 2 and < 2 (%)** | **CKD stage ≥ 2: A, not B (%)** | **CKD stage ≥ 2: B, not A (%)** |
| Cockcroft-Gault vs MDRD4 | 301 (82.9) | 28 (7.7) | 34 (9.4) |
| CKD-EPI vs. Cockcroft-Gault | 313 (86.2) | 11 (3.0) | 39 (10.7) |
| CKD-EPI vs. MDRD4 | 329 (90.6) | 0 (0.0) | 34 (9.4) |
| Cystatin C vs MDRD4 modified* | 280 (77.1) | 80 (22.0) | 3 (0.8) |
| CKD-EPI modified* vs. CKD-EPI cystatin C^a^ | 279 (76.9) | 2 (0.6) | 82 (22.6) |
| CKD-EPI vs. CKD-EPI cystatin C^a^ | 281 (77.4) | 9 (2.5) | 73 (20.1) |
| CKD-EPI vs. CKD-EPI cystatin C^b^ | 305 (84.0) | 12 (3.3) | 46 (12.7) |
| Cockroft-Gault vs. CKD-EPI cystatin C^b^ | 295 (81.3) | 31 (8.5) | 37 (10.2) |
| MDRD4 vs. CKD-EPI cystatin C^b^ | 289 (79.6) | 37 (10.2) | 37 (10.2) |

* with factor for black Americans
^a^ CKD-EPI equation: eGFR = 76.7 x CystC-1.19
b CKD-EPI equation: eGFR = 127.7 x CystC^-1.17^ x age^-0.13^ x 0.91_[if female]_ x 1.06_[if black]_
